# Newborn Screening for X-Linked Adrenoleukodystrophy in Nebraska: Initial Experiences and Challenges

**DOI:** 10.3390/ijns8020029

**Published:** 2022-04-26

**Authors:** Craig V. Baker, Alyssa Cady Keller, Richard Lutz, Karen Eveans, Krystal Baumert, James C. DiPerna, William B. Rizzo

**Affiliations:** 1 Munroe-Meyer Institute for Genetics and Rehabilitation, University of Nebraska Medical Center, Omaha, NE 68198, USA; cbaker@unmc.edu (C.V.B.); alyssa.cady@unmc.edu (A.C.K.); rlutz@unmc.edu (R.L.); 2 Nebraska Newborn Screening Program, Department of Health and Human Services, Lincoln, NE 68509, USA; karen.eveans@nebraska.gov (K.E.); krystal.baumert@nebraska.gov (K.B.); 3 Perkin Elmer Genetics, Pittsburgh, PA 15275, USA; james.diperna@perkinelmer.com; 4 Department of Pediatrics and Child Health Research Institute, University of Nebraska Medical Center, Omaha, NE 68198, USA

**Keywords:** adrenoleukodystrophy, adrenomyeloneuropathy, newborn screening, X-ALD, *ABCD1*, peroxisomal fatty acid oxidation

## Abstract

X-linked adrenoleukodystrophy (X-ALD) is a neurodegenerative disease caused by pathogenic variants in *ABCD1* resulting in defective peroxisomal oxidation of very long-chain fatty acids. Most male patients develop adrenal insufficiency and one of two neurologic phenotypes: a rapidly progressive demyelinating disease in mid-childhood (childhood cerebral X-ALD, ccALD) or an adult-onset spastic paraparesis (adrenomyeloneuropathy, AMN). The neurodegenerative course of ccALD can be halted if patients are treated with hematopoietic stem cell transplantation at the earliest onset of white matter disease. Newborn screening for X-ALD can be accomplished by measuring C26:0-lysophosphatidylcholine in dried blood spots. In Nebraska, X-ALD newborn screening was instituted in July 2018. Over a period of 3.3 years, 82,920 newborns were screened with 13 positive infants detected (4 males, 9 females), giving a birth prevalence of 1:10,583 in males and 1:4510 in females. All positive newborns had DNA variants in *ABCD1*. Lack of genotype-phenotype correlations, absence of predictive biomarkers for ccALD or AMN, and a high proportion of *ABCD1* variants of uncertain significance are unique challenges in counseling families. Surveillance testing for adrenal and neurologic disease in presymptomatic X-ALD males will improve survival and overall quality of life.

## 1. Introduction

X-linked adrenoleukodystrophy (X-ALD) is an inherited neurodegenerative disease caused by pathogenic variants in *ABCD1* that result in impaired peroxisomal beta-oxidation and progressive demyelinating symptoms [1,2]. The disease has a wide clinical expression and age of onset. Approximately 35% of diagnosed X-ALD males exhibit the most severe phenotype known as childhood cerebral X-ALD (ccALD), which is characterized by inflammatory demyelination affecting the central nervous system. Affected ccALD boys are normal at birth but develop neurologic symptoms later in childhood with a median age at diagnosis of 7.0 years [3]. Initial neurologic symptoms may manifest as clumsiness, poor school performance, new-onset attention deficit-hyperactivity, emotional lability, visual decline, or gait abnormalities. Once neurologic symptoms appear, they progress rapidly over several years to a fatal outcome. Older males who manifest the adrenomyeloneuropathy (AMN) phenotype account for about 60% of X-ALD cases [1,2]. Affected AMN males develop spastic paraparesis, peripheral neuropathy, and sphincter dysfunction that typically begins after the second decade of life and worsens over many years [4]. A significant proportion of AMN men ultimately transition to a cerebral demyelinating phenotype similar to boys with ccALD [4,5].

Primary adrenal insufficiency occurs independently of neurologic symptoms in X-ALD. Adrenal involvement can appear as early as several months of age or as late as the 6th–7th decade of life. Most boys with ccALD have overt adrenal insufficiency at the time neurologic symptoms are apparent [6,7]. Unrecognized adrenal insufficiency can prove fatal in children or adults, but is easily treated with adrenal hormone replacement, if detected.

Phenotypic variation in X-ALD is a confounding feature of this disease. Males with ccALD and AMN are often present in the same family, indicating that the *ABCD1* pathogenic variant does not strictly determine the X-ALD phenotype. Further, up to 88% of heterozygous females for the X-ALD gene develop mild AMN-like symptoms of myelopathy and/or peripheral neuropathy by the 6th decade of life, but rarely exhibit adrenal dysfunction or cerebral demyelination [8]. This striking clinical variation has led to unsuccessful searches for modifier genes or other biomarkers to predict X-ALD phenotype [9,10].

Progressive neurologic disease in boys with ccALD can be halted if treated with allogeneic hematopoietic stem cell transplantation (HSCT) at the earliest onset of demyelination [11,12,13] and lentiviral-mediated ex vivo gene therapy is under investigation as an alternative option [14,15]. However, once neurologic symptoms become entrenched, these therapies are ineffective, which underscores the critical need for early diagnosis. Given the known risks associated with HSCT, this procedure is reserved for patients who show evidence of advancing cerebral demyelination. Current recommendations for monitoring presymptomatic boys include regular adrenal hormone testing to detect and treat adrenal insufficiency [7], and serial neuroimaging to identify the early appearance of white matter disease for referral for HSCT [3,16]. In the absence of a family history of X-ALD or newborn screening, the diagnosis of ccALD and AMN is often delayed due in part to its rare nature and insidious neurologic symptoms.

Defective peroxisomal beta-oxidation in X-ALD leads to the accumulation of very long-chain fatty acids (VLCFA). In males, elevated plasma C26:0 and the ratio of C26:0/C22:0 are commonly used as a reliable diagnostic test for X-ALD and are abnormal from birth [17,18]. Owing to variation in X-chromosome inactivation, about 80–85% of female heterozygotes also have increased plasma C26:0. The demonstration of elevated C26:0-lysophosphatidylcholine (C26:0-LPC) in dried blood spots (DBS) opened the possibility of identifying X-ALD infants at the time of birth [19,20,21]. A population-wide newborn screening program first began in New York state in December 2013 [22] and other states soon followed [23,24,25,26,27]. This initiative now allows X-ALD infants to be identified and closely monitored from birth, which promises to save lives by prompt initiation of therapy and redefine the true natural history of the disease.

Newborn screening for X-ALD started in Nebraska on 1 July 2018. Here, we summarize our initial experience with screening infants at birth and counseling families about the unique aspects of this disease.

## 2. Materials and Methods

The state of Nebraska contracts with PerkinElmer Genetics. (Pittsburgh, PA, USA) to perform newborn screening for X-ALD. Of note, in Nebraska newborn screening is compulsory and no exceptions are allowed. Infants born at home are also required to be screened. Guidelines dictate that all newborns are screened at 24–48 h, or prior to hospital discharge if released before 24 h of age. Screens drawn before 24 h of life are to be repeated by 7 days of life and premature infants in the Neonatal Intensive Care Unit have a repeat screen at 28 days of age.

### 2.1. Dried Blood Spot Analysis

A 1/8″ punched DBS was extracted in methanol at room temperature with 31 picomoles deuterated-C26:0-LPC (Avanti Polar Lipids, Inc., Alabaster, AL, USA) as an internal standard. The extract was diluted 70/30 with HPLC-grade acetonitrile/water in 0.6% formic acid and analyzed by tandem mass spectrometry (MS/MS) using electrospray ionization in positive ion mode. In order to eliminate an interfering compound with the same mass as C26:0-LPC, a two-tiered testing process was utilized to detect X-ALD newborns.

1. Tier 1 screening: C26:0-LPC was measured using flow injection tandem mass spectrometry (FIA-MS/MS). The C26:0-LPC cutoff was 0.36 µmol/L.
2. Tier 2 screening: C26:0-LPC was measured by high-pressure liquid chromatography-MS/MS using a Waters Xterra C8 LC column inserted between the autosampler and mass spectrometer. The cutoff for C26:0-LPC was 0.15 µmol/L.

Screening Algorithm: For newborns with first-tier C26:0-LPC concentration above the cutoff, tier 2 testing on the same initial DBS was done. If the 2nd tier C26:0 LPC was above the cutoff, a second specimen was obtained from the newborn, and repeat tier 2 testing was done. If the C26:0-LPC concentration was still above the cutoff on the repeat specimen, *ABCD1* sequencing was performed. Gene-specific long-range PCR was completed to capture the genomic sequences for the *ABCD1* gene. Next-generation sequencing was performed on an Illumina system with 100 base pair paired-end reads. Variants for the *ABCD1* gene were evaluated using the Adrenoleukodystrophy Variant Database (https://adrenoleukodystrophy.info, accessed on 8 January 2022) in addition to the Human Gene Mutation Database (www.hgmd.cf.ac.uk, accessed on 8 January 2022) and published literature. Variants were also evaluated by their frequency as reported in public databases, (e.g., gnomAD.broadinstitute.org).

### 2.2. State Coordination of Positive Report

Abnormal results on the initial blood spot were reported by PerkinElmer Genetics to the newborn screening coordination team at the Nebraska Department of Health and Human Services. If screening was abnormal on the first DBS, the newborn screening coordinator contacted the primary care provider to request a second heel-stick DBS be submitted. If tier 2 screening remained positive on the second DBS, reflex *ABCD1* gene sequencing was initiated. When *ABCD1* sequencing was completed, the on-call metabolic specialist was notified by the screening coordinator of all results. Infants with positive test results were subsequently seen in the Inherited Metabolic Disease Clinic at Children’s Hospital and Medical Center in Omaha. Both male and female newborns with a positive screen were followed up. Confirmatory biochemical testing for total plasma VLCFAs was typically done together with red blood cell plasmalogen testing to rule out a peroxisomal biogenesis disorder, especially if *ABCD1* sequencing was not definitive or the newborn was not from a known X-ALD family. The clinic genetic counselor met with the parents during the initial visit to obtain a detailed family history, discuss family implications of screening results, and coordinate *ABCD1* variant cascade testing (as needed).

## 3. Results

### 3.1. X-ALD Newborn Screening Outcomes

A total of 82,920 newborns were screened in Nebraska between 1 July 2018 and 31 October 2021 (42,332 males and 40,588 females). Newborn filter cards with unknown/undocumented gender were categorized based on cross-matching with their birth certificate. After testing the initial DBS, 23 newborn screens (9 males and 14 females) were flagged as positive with an elevated C26:0-LPC. Follow-up specimens were obtained after a mean time of 6.3 days and repeat screens confirmed elevated C26:0-LPC in five males and nine females (Table 1). However, one male newborn was premature and had a normal C26:0-LPC on day 3 of life, but mildly elevated C26:0-LPC on day 28. Follow-up plasma VLCFA testing and RBC plasmalogens were normal, and his *ABCD1* sequence was normal, indicating a false positive. Of note, false-positive newborns had a lower mean tier 2 C26:0-LPC level (mean 0.18, range 0.15–0.28) on the first DBS than true positive newborns (mean 0.56, range 0.27–0.90) (*p* = 0.0003), and most false positives (5/9) were at the C26:0-LPC cutoff (0.15 µmol/L). Thus, 13 infants were confirmed as positive on newborn screening. The positive predictive value after testing the second DBS specimen was 80% in males and 100% in females.

In our cohort, the birth prevalence of all true positive newborns was 1:6378. The birth prevalence in males was 1:10,583 and in females was 1:4510. Except for one male with Hispanic ancestry, all positive newborns were Caucasian.

Positive newborns with variants of uncertain significance (VUSs) and a negative X-ALD family history had RBC plasmalogens measured to screen for peroxisomal disorders. No newborn with a peroxisome biogenesis disorder was detected.

### 3.2. Genetic Findings

*ABCD1* sequence variants were identified in all 13 positive cases (Table 1). Ten different variants were documented. Of these, missense variants accounted for all but one: a novel in-frame 3-bp deletion (c.1586_1588del; p.Gly529del). Four of the sequence variants were not listed in the ALD Variant Database.

Of all positive newborns, 9/13 (69%) carried a pathogenic variant based on software predictions, classification in the ALD Variant Database, or a known family history of X-ALD. Two female newborns were cousins who carried the same c.630C > G (p.His210Gln) *ABCD1* variant and two apparently unrelated newborns carried c.1747G > A (p.Val583Met). Two additional newborns with the c.873G > C (p.Glu291Asp) variant were related to a previously known X-ALD family. VUSs were identified in 4/13 (31%) newborns, two males, and two females. One novel missense variant, c.1573C > A (p.Pro525Thr), is classified as a VUS but the same amino acid (Pro525) is replaced with Ser or Ala in the ALD Variant Database and listed as pathogenic or likely pathogenic, respectively.

Maternal inheritance of the *ABCD1* variants was documented in 7/13 (54%) of newborns. Paternal inheritance was confirmed in 2/9 (22%) female cases. *De novo* missense variants were identified in 2/13 (15%) cases, all were female heterozygotes. A third newborn variant is presumed *de novo* based on normal VLCFA analysis in both parents and normal *ABCD1* variant testing in the mother, but DNA testing was not obtained in the father.

A family history of X-ALD or a family history highly suspicious for neurologic disease in males was elicited in 6 (46%) of the newborn cases. Eliminating families with known X-ALD (*n* = 2) and *de novo* cases (*n* = 3), we found 26 newly identified at-risk males among 1st degree to 3rd degree relatives.

### 3.3. Subsequent Follow-Up Care

Four X-ALD males were identified and longitudinal follow-up was recommended after the initial clinic visit. In one case, the parents have refused subsequent follow-up, despite having a maternal family history suggesting possible X-ALD individuals and repeated attempts at education. The other three male newborns have continued to be monitored by our clinical service with normal screening for adrenal insufficiency. All are developing normally. The oldest male with established longitudinal care is almost 2 years old.

Nine X-ALD females were identified by newborn screening. This included one case from a known X-ALD family in which there was prenatal molecular confirmation of a pathogenic variant. After an initial clinic visit to evaluate the need for familial cascade testing and provide genetic counseling, the parents of these female newborns were recommended to pursue follow-ups for their daughters when entering adulthood, for genetic counseling on reproductive risks, and education about the possible onset of clinical symptoms.

Among the two females identified who inherited their *ABCD1* variant alleles from their fathers, both fathers were apparently asymptomatic. Although recommended, adrenal hormone testing has not been performed for these at-risk fathers, to our knowledge.

## 4. Discussion

### 4.1. Observed Birth Prevalence(s) and de novo Rate

X-ALD is a relatively recent addition to the Nebraska newborn screening program, implemented to identify presymptomatic males at-risk for both adrenal insufficiency and ccALD. Based on our cohort, the estimated birth prevalence of X-ALD in males is 1:10,583 (4/42,332). This is similar to a recent X-ALD screening outcome reported by California, with a male birth prevalence of 1:10,504 (90/945,344) [26]. However, we note this birth prevalence is not explicitly reported, as California increased their tier 2 C26:0-LPC cut-off 1.8 years into their study period, which decreased the observed birth prevalence in males from 1:7181 to 1:14,390. The observed male birth prevalence of 1:10,583 in our Nebraska cohort is less than reported by Minnesota (1:3800) [23] and North Carolina (~1:8800) [25].

Other reports on newborn screening for X-ALD in some states do not differentiate birth prevalence based on biological sex. Therefore, comparing our overall birth prevalence of 1:6378, the observed Nebraska birth prevalence remains less than Minnesota (~1:4845) [23] but greater than reported by New York (~1:14,700) [26], Georgia (1:51,081) [24], North Carolina (1:8717) [25], California (~1:11,500 using current C26:0-LPC cut-off) [26], and Illinois (~1:16,200) [27]. Our comparably higher birth prevalence may reflect an ethnically more homogenous population, founder effects, methodological differences in laboratory analysis of the DBS, and cutoff values. By mandating screening of all newborns for X-ALD in Nebraska, racial disparities in the diagnosis of this disease are eliminated [28].

Interestingly, in our relatively small cohort at least two and possibly three of thirteen (23%) X-ALD newborns, all female, were found to represent *de novo* cases. This *de novo* rate appears higher than expected, perhaps due to the small number of newborns detected. In contrast, the *de novo* rate of X-ALD index cases has been previously estimated at 4.1% based on molecular studies of 489 X-ALD families ascertained by symptoms [29].

We do not know the false-negative rate for X-ALD newborn screening, but two females born to known X-ALD families were appropriately detected. To date, we have detected at least twice as many females on newborn screening compared to males, suggesting the screening test is highly sensitive.

### 4.2. Experiences with Follow-Up Care and Genetic Counseling

An inevitable outcome of X-ALD newborn screening is the identification of families that have male individuals with previously undiagnosed neurologic disease. The news of positive newborn screening results can lead to difficult conversations among family members. Of our X-ALD newborns, 6/13 (46%) had a family history that was suggestive of X-ALD in male relatives or known to be positive for X-ALD. The wide clinical variation in age of onset and severity of X-ALD symptoms underscores the value of family studies for identifying at-risk individuals and may end a family’s diagnostic odyssey.

Despite the promise to save additional lives of at-risk family members, newborn screening for X-ALD presents several unique challenges in counseling families [30]. Since phenotype cannot be informed by genotype or severity of biochemical abnormalities, families of males with X-ALD are placed in the stressful position of “anticipatory anxiety” during the first decade of life while their child is monitored with serial MRIs and adrenal testing. It is common for parents to be hypervigilant about any unusual behavior or misstep by their son. Until a reliable predictive biomarker is found to distinguish ccALD from later-onset AMN, this extra burden of family anxiety will not be relieved. Additionally, despite the potential for some degree of reassurance if a male child escapes the typical age range of ccALD presentation, the anticipation of AMN neurologic symptoms with no effective treatment remains.

In addition to anxiety caused by uncertainty about X-ALD symptom onset, some families have other negative reactions to receiving X-ALD newborn screening results [30]. For example, in one case the family history revealed male relatives affected by symptoms consistent with X-ALD, but an influential family member denied that the newly diagnosed infant could have X-ALD. This family has refused subsequent follow-up, despite multiple efforts by our team. In another case, targeted variant testing was obtained for an older teenage brother who was found to be positive after assenting but has refused follow-up clinical evaluation for fear that abnormal findings will interfere with his interest in pursuing a military career.

Like other states that have implemented newborn screening for X-ALD, Nebraska reports all positive female cases. This differs from The Netherlands where only X-ALD males are tested [31]. The ability to identify at-risk family members is hindered if X-ALD females are not reported. Because X-ALD females do not develop symptoms in early life, counseling these families is reassuring them that there is no immediate medical impact on their infant. However, it is critical to maintain contact with the families to reinforce education about genetic implications for family planning and the likelihood that the X-ALD female will develop an AMN-like phenotype in late adulthood.

### 4.3. Variants of Uncertain Significance

We encountered several VUSs in our newborn cases, which presented another challenge for counseling because of less certainty that a VUS will lead to future X-ALD symptoms. Although all positive newborns have displayed biochemical evidence of impaired VLCFA metabolism, it is still possible that an unknown number of X-ALD infants identified at birth may never become symptomatic or only have mild subclinical disease. This conundrum underscores the importance of longitudinally following these families and incorporating new clinical data into public databases, (e.g., the ALD Variant Database [32]).

### 4.4. Future Outlooks

The ability to perform newborn screening for X-ALD is expected to lead to major advances in understanding the natural history of this disease. The identification and monitoring of X-ALD newborns as they age through childhood offers the opportunity to discover predictive biomarkers unique to ccALD or AMN. Realization of these benefits will require collaborative efforts across states to develop a registry with clinical data and a biobank of specimens for future investigations.

## 5. Conclusions

Our initial experience with newborn screening for X-ALD has proven its efficacy for detecting X-ALD infants and identifying families with at-risk individuals, but it is not without its challenges with respect to genetic counseling, education, and patient compliance. The contributions of geneticists, neurologists, and endocrinologists will be needed for the optimal care of these patients. As the first cohort of newborns is followed in the coming years, the significant impact of newborn screening for X-ALD patients will become increasingly recognized.

## Figures and Tables

**Table 1:** Nebraska newborns detected as true positive cases of X-ALD (1 July 2018–31 October 2021).

Male/Female	C26:0-LPC (Tier 2)	*ABCD1* Variant	Variant Interpretation	Cataloged in X-ALD Database?	Inheritance	Relatives with Neurologic Disease or X-ALD?
M	0.26	c.1600C > T (pPro534Ser)	Pathogenic	Yes	Maternal	Yes
M	0.26	c.1747G > A (p.Val583Met)	Uncertain Significance	Yes—Status Unknown	Maternal	No
M	0.90	c.873G > C (p.Glu291Asp)	Pathogenic *	Yes	Maternal	Yes, X-ALD
M	0.58	c.1586_1588del (p.Gly529del)	Uncertain Significance	No	Maternal	No
F	0.37	c.1573C > A (p.Pro525Thr)	Uncertain Significance	No **	Not Confirmed	Yes
F	0.23	c.630C > G (p.His210Gln)	Pathogenic ***	No	Maternal	Yes
F	0.25	c.887A > G (p.Tyr296Cys)	Pathogenic	Yes	*De novo*	No
F	0.45	c.1028G > A (p.Gly343Asp)	Pathogenic	Yes	Paternal	No
F	0.30	c.630C > G (p.His210Gln)	Pathogenic ***	No	Maternal	Yes
F	0.37	c.2006A > G (p.His669Arg)	Pathogenic	Yes	Presumed *De novo*	No
F	0.23	c.1747G > A (p.Val583Met)	Uncertain Significance	Yes—Status Unknown	Paternal	No
F	0.20	c.1534G > A (p.Gly512Ser)	Pathogenic	Yes	*De novo*	No
F	0.79	c.873G > C (p.Glu291Asp)	Pathogenic *	Yes	Maternal	Yes, X-ALD

* Genetically related to a known X-ALD family. ** Although p.Pro525Thr is not in the ALD Variant Database, replacement of Pro525 with Ser or Ala is classified as pathogenic or likely pathogenic, respectively. *** These newborns are known to be related as maternal cousins.

## Data Availability

Data in this study are available by request from the corresponding author.
